# Validity Beyond Measurement: Why Psychometric Validity Is Insufficient for Valid Psychotherapy Research

**DOI:** 10.3389/fpsyg.2019.00532

**Published:** 2019-03-12

**Authors:** Femke L. Truijens, Shana Cornelis, Mattias Desmet, Melissa M. De Smet, Reitske Meganck

**Affiliations:** Faculty of Psychology and Educational Sciences, Department of Psychoanalysis and Clinical Consulting, Ghent University, Ghent, Belgium

**Keywords:** validity, epistemic validity, validity or research, psychotherapy research, evidence-based treatment, empirical case study, evidence-based case study

## Abstract

In psychotherapy research, “validity” is canonically understood as the capacity of a test to measure what is purported to measure. However, we argue that this psychometric understanding of validity prohibits working researchers from considering the validity of their research. Psychotherapy researchers often use measures with a different epistemic goal than test developers intended, for example when a depression symptom measure is used to indicate “treatment success” (cf. outcome measurement for evidence-based treatment). However, the validity of a measure does not cover the validity of its *use* as operationalization of another target concept within a research procedure, nor the validity of its function toward an epistemic goal. In this paper, we discuss the importance of considering validity of the epistemic process beyond the validity of measures *per se*, based on an empirical case example from our psychotherapy study (“SCS”, Cornelis et al., 2017). We discuss why the psychometric understanding of validity is insufficient in covering epistemic validity, and we evaluate to what extent the available terminology regarding validity of research is sufficient for working researchers to accurately consider the validity of their overall epistemic process. As psychotherapy research is meant to offer a sound evidence-base for clinical practice, we argue that it is vital that psychotherapy researchers are able to discuss the validity of the epistemic choices made to serve the clinical goal.

## Introduction

Any psychology scholar looking for information on the validity of a psychotherapeutic or clinical study knows where to find it: under “Measures” in the Methods section. In psychotherapy research it is common, and often formally required for publication, to use the IMRAD-format (Introduction-Methods-Results-and-Discussion) to report on empirical results, in which the use of validated instruments^1^ is presented in the Methods section (cf. Madigan et al., 1995). However, in this paper we argue that the heuristic placement of validity issues under the Measures header gives the false impression that validity only matters with regard to measures. As psychotherapy research is applied research with the clear goal of understanding and improving clinical practice, the validity of the *entire* research process is vital for epistemic, clinical and societal reasons. With this paper, we aim to address psychotherapy researchers and use concrete clinical research data to discuss why it is insufficient for valid psychotherapy research to limit the understanding of validity to instruments.^2^

In psychological research, validity is generally understood as a psychometric concept that refers to “measur[ing] what is purported to measure” (Borsboom et al., 2004, p. 1061). Newton and Shaw (2013, p. 203) note that in different scientific fields validity may have a broader scope than just measurement issues, yet still they limit their discussion to the psychometric definition of validity, as do the majority of scholars that are currently involved in the debate (e.g., Crooks et al., 1996; Kane, 2001, 2013; Borsboom et al., 2004; Hood, 2009; Cizek, 2012). In this paper, we start from the observation that the use of the term validity in psychotherapy research is predominantly understood in psychometric terms. This might be less of a problem if test construction is the sole goal of research, but we argue that it is highly problematic in the broader scientific endeavor of *applied* research such as psychotherapy research.

Applied research can be distinguished from fundamental or basic research. Applied research is focused at gaining knowledge with the explicit goal to apply this knowledge in non-scientific contexts, rather than to gain knowledge for the sake of knowledge expansion *per se* (cf. Danziger, 1990). For example, psychotherapy research^3^ is conducted to be able to disseminate “evidence-based” treatments into clinical practice, technical innovation or artificial intelligence research can be focused at improving concrete daily tasks or societal systems, educational research is focused at *in situ* assessment within the various learning environments, environmental research can be conducted with the explicit goal to design political policy, et cetera. Practically, this goal-oriented base of applied research implies that local, historical and social circumstances may play a substantial role in the research procedure (cf. Douglas, 2009). Whereas such factors may be the *object of interest* in fundamental research, in applied research they may also play an important role from the decision to study a specific topic all the way to the decisions on how to apply, whom to disseminate findings to and on what scale, what impact the application may have, et cetera (cf. Cartwright and Stegenga, 2011, on evidence generation focused on *use*).

In applied research, to design a methodologically sound study, researchers thus have to make a broad range of epistemic choices before and beyond measurement. However, as “validity” is heuristically used with reference to instruments, these vital epistemic choices cannot properly be judged on their validity because they do not fall under instrumental validity *per se*. Therefore, we argue that it is crucial for psychotherapy research to *be able to think about validity in a broader epistemic sense* than the current focus on test validity allows for.

The problem that we address in this paper is not new. The question of what constitutes valid research is central to on-going discussions in psychotherapeutic and methodological research literature, that yield the problem of operationalization (e.g., cf. Danziger, 1990; Westen et al., 2004; Barkham et al., 2010; Castonguay, 2011), the nature of psychological constructs and variables (e.g., Michell, 1997; cf. Woodward, 1989; Toomela, 2007) and how accurately they are represented by measures (Michell, 2013; Tal, 2016; cf. Truijens et al., 2019), the issue of choosing primary outcome variables (Wampold, 2001; De Los Reyes et al., 2011) and according measures (e.g., US Department of Health and Human Services Food and Drug Administration, 2009), and how this choice affects the interpretation of outcome (Stegenga, 2015), the discussion on qualitative or quantitative methods of analysis (e.g., McLeod, 2001; Stiles, 2006; Hill et al., 2013; Gergen et al., 2015), the discussion on clinical significance (Jacobson and Truax, 1991; Lambert and Ogles, 2009), and so on. However, as these issues are discussed in different corners of psychological, methodological, medical and philosophical literature, it often goes unnoticed that they all fall under the broader question of validity of the epistemic process of psychotherapy research. A variety of issues are thus known and discussed in their own terms, but the field lacks a meta-theory or conceptual framework to consider how these issues connect, thus indicating a common root in the overall epistemic process. This leads to the underestimation of the impact of these epistemic issues and prevents working researchers from properly taking these issues into consideration in their scientific endeavor. In other words: because of a lacking conceptual framework that is broad enough to encompass the shared roots of the issues voiced in the literature, researchers cannot take the problems seriously *enough*.

Even though the voiced critiques and worries are substantive and persuasive, they apparently have not sufficiently reached working psychologists. The aim of our paper therefore is straightforward: we want to show as clearly as possible why test validity is insufficient in capturing the validity of the overall research endeavor in psychology, to concretely imply awareness in working psychologists. We use the working concept *validity of the epistemic process*, or – in short – epistemic validity, to elaborate and denominate the connection between the various epistemic problems voiced in the literature, and to allow for concrete consideration of their impact on the validity of conducted psychotherapy research. This way, we argue for the need of a conceptual framework of epistemic quality control that can broaden the classic IMRAD-format such that the issues faced in designing and conducting psychotherapy research can be discussed as thoroughly as needed, given their substantial impact on the understanding of psychotherapeutic efficacy and clinical practice.

## Operationalization and Test Validity in Psychotherapy Research

**FIGURE 1:**
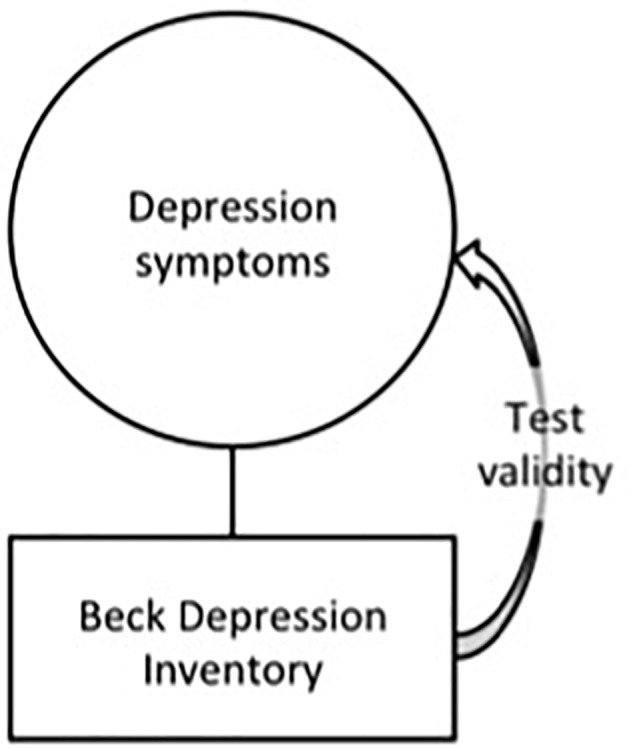
The Beck Depression Inventory (BDI) graphically displayed as a valid operationalization of the construct depression symptoms.

In this section we first argue that in psychotherapy research, validity is and should be understood more broadly than test validity alone, which we illustrate subsequently with a case from our psychotherapy study. We start our argument using the Beck Depression Inventory (BDI; Beck et al., 1996) as an exemplar. The BDI is a very commonly used instrument to detect depression symptoms as defined by the DSM-IV (Rogers et al., 2005). The validity of the instrument was tested in a multitude of studies and was summarized by Beck et al. (1988). The test has detection of depression symptoms as its *end*, and the test validity confirms that the instrument is adequate as a *means* to satisfy the proposed end. Consequently, the measure can serve as a valid operationalization of the construct it aims to measure.^4^ This relationship between construct and instrument is graphically displayed as in Figure 1. Note that in this figure we used the graphics that are common in psychological education: The circle represents the construct or variable and the square represents the operationalization of that construct (Mook and Parker, 2001).

In practice, the BDI is indeed commonly used in the context of clinical diagnostics (Rogers et al., 2005). Beyond such direct depression symptom detection, however, the BDI is also increasingly used as a so-called “outcome measure” in psychotherapy research. Outcome measures are used to gain systematic quantified evidence on the efficacy of treatments in samples of patients, which is becoming an increasingly common practice in the era of evidence-based treatments (EBTs; Wampold, 2001). Practically, instruments such as the BDI are administered to a sample of patients before and after a specific treatment, and the pre-post difference score of this sample is compared to a sample of patients who did receive different or no treatment (ibid.). In this case, the BDI is used to indicate depression severity changes over the course of a treatment, which is used as an indicator of the efficacy of the treatment that was administered.

**FIGURE 2:**
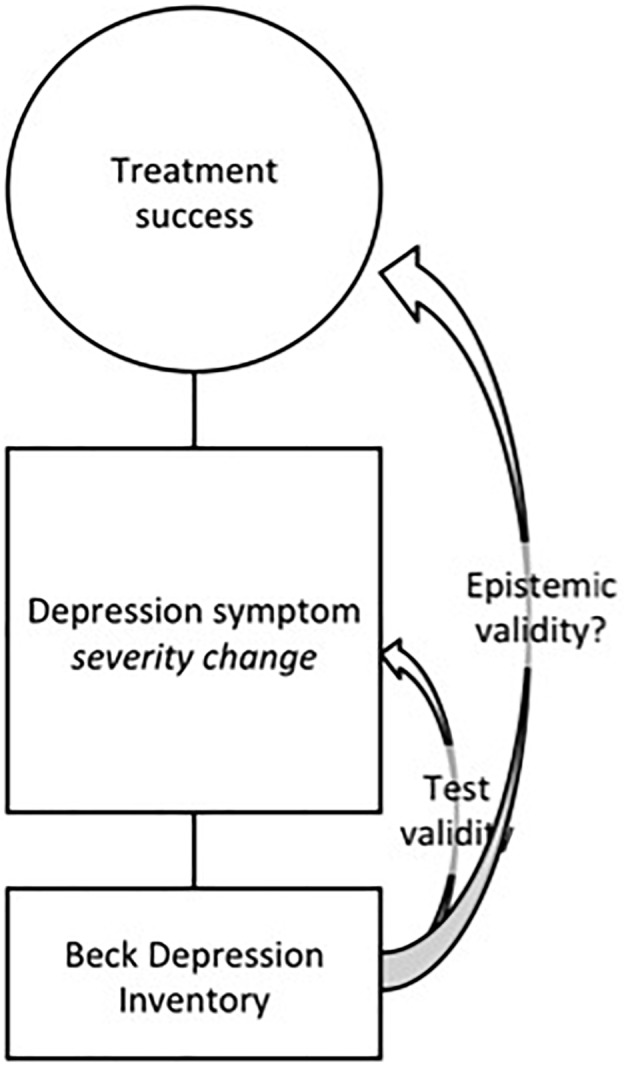
Treatment success is operationalized as depression symptom severity change, which is operationalized as a difference score on the BDI (Beck et al., 1988).

In this research context, the BDI serves as the operationalization of the concept “treatment efficacy”. Consequently, the BDI is no longer simply the operationalization of the concept of “depression symptoms”, but it becomes the operationalization of the concept “depression severity change over time”,^5^ which itself functions as the operationalization of the concept “treatment efficacy”. This sequence of operationalizations is shown in Figure 2, where the additional operationalization “change in depression symptoms severity” is displayed in a square between the concept “treatment success”^6^ and the operationalization “BDI”. In this design, the BDI is used as the instrument to indicate treatment success, even though an additional step was added to the operationalization sequence. As the target concept of the BDI now becomes an operationalization of another concept, namely “treatment success”, the BDI serves as the means toward *another end* than its initial end of simple depression symptom detection.

At this point, however, the question rises whether the test validity of the BDI covers this additional step in the sequence of operationalizations. The validity of the BDI serves as an indicator of accurate depression symptom measurement, i.e., the validity of the instrument indicates that the BDI is valid as a means to satisfy its end, which is depression symptom detection. However, this does not necessarily indicate that the BDI is a valid indicator of treatment success, nor that it is inherent to the concept treatment efficacy to operationalize it as symptom severity change as measured by the BDI. As becomes clear from Figure 2, the test validity of the instrument BDI is only *part* of the epistemic validity of the operationalization of treatment efficacy *per se*. Therefore, it is not feasible to rely on the validity of tests as reported in the Measures section, to guarantee the epistemic validity of the overall study design that is embedded in an epistemic procedure by researchers. In the next section, we present an empirical case study to emphasize the importance of epistemic validity for concrete psychotherapeutic research.

### Case: Where Validity Goes Beyond the Validity of Tests

To clarify the relationship between test validity and epistemic validity in the practical context of psychotherapy research, we discuss the findings from an empirical case study by Cornelis et al. (2017),^7^ that was conducted in the context of a broader psychotherapy study that was conducted at Ghent University, Belgium (“SCS”, cf. Desmet, 2018). In the following, we briefly describe the study outline and the research team, and subsequently discuss the case findings, to set the stage for our argumentation in empirical terms.

#### Study Outline

**FIGURE 3:**
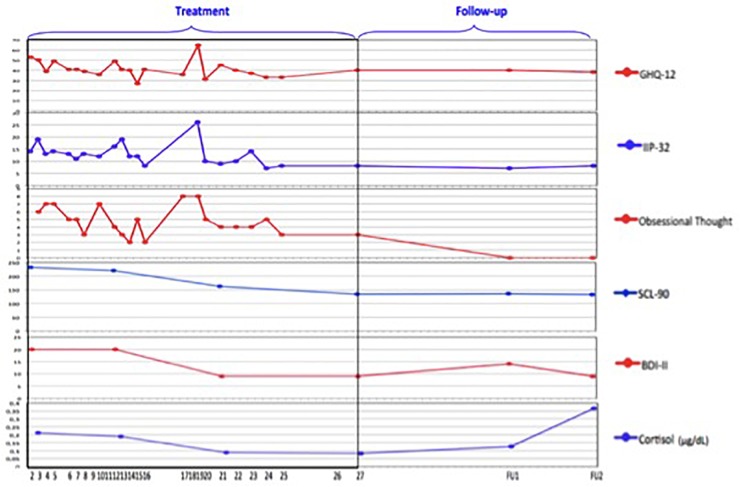
Individual outcome measure battery throughout treatment for patient James (retrieved from Cornelis et al., 2017; reprinted with permission).

The data used in this paper were gathered in a mixed method psychotherapy study conducted at Ghent University from 2009 onward (Cornelis et al., 2017). In this study, patients in a private psychotherapy practice were followed on a session-by-session basis with a variety of means. Every month, patients completed validated symptom measures such as the BDI (Beck et al., 1996), the Symptom Checklist (SCL-90; Derogatis, 1992), and the Inventory of Interpersonal Problems (IIP-32; Horowitz et al., 2000). Every session, patients scored the General Health Questionnaire (GHQ; Goldberg and Williams, 1988) and an idiosyncratic item that was based on the primary complaint at the start of treatment as formulated by the patient. Furthermore, the patient collected saliva samples, which allowed for analysis of cortisol stress hormone development over the course of therapy and follow-up. Every treatment session was audiotaped to enable qualitative and narrative analyses. Patients agreed to participate in four follow-up interviews in the 2 years after treatment termination, which were accompanied by the same test battery and biological data collection as during treatment. Finally, 2 years after termination, patient’s health insurance files were requested, which yielded the health care costs from 2 years before the start of therapy up till 2 years after treatment termination. Figure 3 shows the information gathered for each patient in the study.^8^

#### Research Team

All authors were involved in the conduct of the SCS psychotherapy study, which was supervised by the third and fifth author. The therapy was conducted in the private practice of the third author. At the start of the study, the therapist was a 36-year old Caucasian male with 5.5 years of clinical experience in psychodynamic psychotherapy, based on principles of supportive-expressive treatment as defined by Luborsky (1984). All five authors were employed as researchers at the university department that hosted the study. The first, second, and fourth author were doctoral candidates and were involved in the data collection throughout their terms. Regarding the current case, they were involved in the management of the quantitative and biological data collection and they conducted interviews. A systematic case study was conducted by a research team including the second, third, and fifth author (cf. Cornelis et al., 2017, for a description of the methodological process). For the current paper, the five authors were involved in a reflection on the interpretations of the findings, which was used by the first author to derive a vignette of the case that serves as an empirical exhibit within the argumentation on validity.

#### Case

James started treatment voluntarily after being referred by his general practitioner. After the second preliminary session with his therapist, he agreed to participate in the psychotherapy study. James received 26 sessions of supportive-expressive treatment (cf. Luborsky, 1984). At the start of treatment, James, a Caucasian male, was 29 years old and suffered from depression- and anxiety complaints related to an obsessive thought that started when he met his girlfriend. James was terrified that he would stab his girlfriend with a knife, and that he would not be able to control himself. This brought up a range of life-long fears of being a loser and a harmful person to other people, which he thought made him unworthy of life.

At the start of treatment, James’s BDI score was 20, thus his depression symptom severity could be considered moderate^9^ (Beck et al., 1996). After treatment termination, he only scored 9 on the BDI both at post-measure and at the 2-year follow-up, which would be classified as minimal (ibid.). Based on the BDI as the primary outcome measure (cf. De Los Reyes et al., 2011), this reduction of pre-post scores could be taken as an indication that the therapy has been successful (see Cornelis et al., 2017, for detailed information on individual clinical significance of this reduction). For James, this conclusion is in line with narrative information in the follow-up interviews: although the obsessive thought still popped up every now and then, James felt confident that he could ignore that thought, which significantly reduced his experience of fear. Moreover, as he got the reassurance of control over these fears, he felt worthier of living. Besides the residual anxiety symptoms, he explicitly stated that he did not experience depression anymore. This cross-validation or triangulation indeed indicates a reduction of initial depressive symptoms, and therefore supports a positive conclusion regarding treatment success. Note that it is somewhat simplistic to reach this conclusion based on a single individual pre-post difference, yet it still functions as an illustration of common methodological reasoning in psychotherapeutic research (see Truijens, 2017, for a discussion of this line of reasoning in psychotherapeutic efficacy methodology).

**FIGURE 4:**
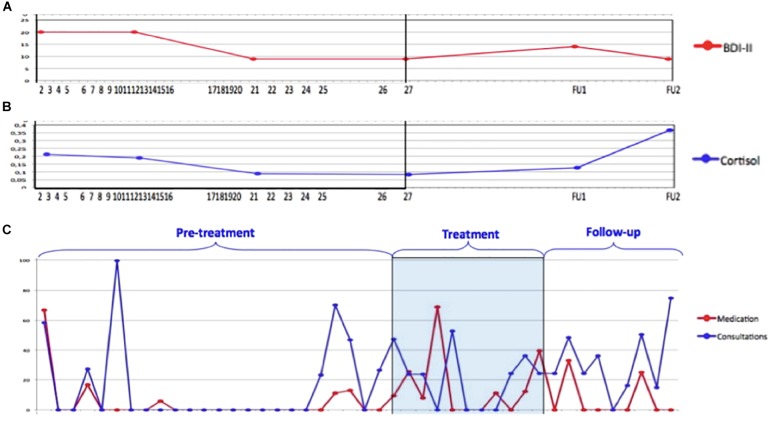
Three different outcome or process measures for patient James (retrieved from Cornelis et al., 2017; reprinted with permission): **(A)** the BDI (Beck et al., 1988) from baseline to 1-year follow-up; **(B)** cortisol stress hormone measures from baseline to 1-year follow-up; **(C)** medical health care costs from years prior to 2 years after treatment.

Whereas the BDI is often used as an outcome measure, within the data collection in this psychotherapy study, several of other data sources could have been used as outcome measures and therefore as operationalizations of treatment success as well. As was discussed above, the BDI shows a symptom reduction that was in line with narrative follow-up information, indicating a long-term impact of the treatment on James’s complaints. However, James’s cortisol levels show a different image of long-term success: whereas his stress levels indeed reduced over the course of treatment, at the second follow-up his stress hormone levels were about twice as high as baseline (Figure 4A,B). This might change our idea of treatment efficacy in the long run, as the stress hormone levels show an important reduction *during* treatment but an alarming increase after treatment, which may impact the long-term durability of treatment success (Cornelis et al., 2017). However, it is not evident to reach a sound and clear conclusion based on this number; to make sense of this increase, the idiosyncratic information should be taken into account to find out whether the increase is related to depressive symptoms or to, for example, regular life stressors (cf. Truijens, 2017).

Nonetheless, these stress hormone levels could be a highly informative operationalization of treatment success, especially because of the long-term health problems such as cardiovascular diseases and metabolic issues that are related to elevated stress levels (Walker, 2007; Wester and Van Rossum, 2015), which are associated with increasing societal and financial risks (World Health Organization, 2008). Thus, for a researcher who was interested in the societal cost-benefit balance this could be an interesting way to operationalize treatment success. If this operationalization was used as the primary outcome measure, the conclusion of treatment success in this case would be rather ambiguous in the long run and would at least need more information to understand the stress level increase.

A second alternative source of information on the treatment success in James’s case could be the information on his medical health costs (Figure 4C). This shows both his number of consultations with general and medical practitioners and his medication use, which in James’s case was anti-depressant medication. Whereas for James, his reported depression symptoms changed on the BDI, the dose of anti-depressant drugs was increased for example quite shortly after treatment termination. If this data source was taken as the primary outcome measure, the image of “treatment success” would be rather different than if we would take self-report information on the BDI and in follow-up interviews as our primary outcome measures.

The two examples of alternative outcome measures show either a more ambiguous or an entirely different story of treatment success than the BDI does as the primary outcome measure. This could spark a discussion on the convergent or concurrent validity of these measures, which would ultimately lead to a discussion on the construct validity of each of these means as satisfying the end of treatment success indication. However, by tapping into the discussion of test validity right away, we would skip a rather crucial step in the empirical process that comes before the question of construct validity of measures.

The previously discussed “outcomes” in James’s case show three possible ways of operationalizing “treatment success”. These three ways are *ad hoc*, as they entail the measures that *we* – the authors – as psychotherapy researchers decided to use in our study design, given our theoretical, empirical and clinical framework and our clinical and epistemic goal (cf. Cornelis et al., 2017). Importantly, this shows that the operationalization that is chosen by a researcher, a group of researchers, or a paradigmatic field of researchers, *is not self-evident*. There is no inherent reason to choose a specific operationalization in applied research designs such as the one discussed here in psychotherapy research. There is no inherent characteristic or ontological essence in a concept such as “treatment success”.^10^ So before asking whether a specific measure is valid in doing its job as an indicator of such a target concept, the researcher must decide how he intends to operationalize that concept in the first place.

This notion has a rather crucial consequence for psychotherapy researchers: Regardless of the validity of the BDI as a means to indicate depression symptom severity, the researcher should be able to validate his decision to operationalize the target concept “treatment success” as “depression symptom severity change” (cf. Westen et al., 2004, for a discussion of this specific operationalization). So, before the researcher asks the question of validity of his instruments, he must first decide *which* means to use to satisfy *his* proposed operationalization. Surely, this decision must be informed by the validity of different available means – such as the BDI but also the cortisol levels or the health insurance costs – but first the researcher should be able to show that his proposed operationalization is valid to satisfy its proposed end – such as indicating “treatment success”.

**FIGURE 5:**
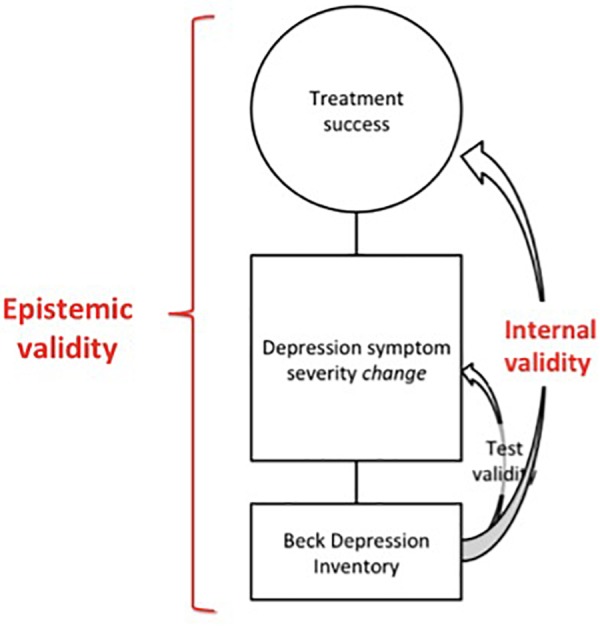
The validity of the research process entails both the internal validity of the design and the epistemic validity of the function of the design in a researcher’s epistemic endeavor.

The crucial point therefore is that it is a *choice* by the researcher how he or she operationalizes the concept of interest. This choice may be informed by theory, by common epistemic practice, by experience, by face validity, et cetera. Either way, it is an epistemic choice in which the researcher plays a crucial role. Therefore, in the case of James we could question the way treatment success was operationalized in general, and more importantly we could ask which way of operationalizing is more useful for the purposes of that specific research. Especially in the era of EBT, this epistemic pragmatism is a very relevant question for psychotherapy research: All research designs are chosen in function of *some epistemic context* that makes it relevant and important to conduct that particular study (Figure 5).

As becomes clear from Figure 5, neither the way that a concept is operationalized nor the function of the process of operationalization in the overall epistemic endeavor of the researcher are (necessarily) covered by the test validity of the instrument that is used. Nonetheless, these steps generate real and relevant validity questions.^11^ To capture this broader epistemic context in which the research design is embedded, we use the working concept epistemic validity.^12^ In the next section, we argue that this understanding of validity goes beyond the current validity debates in the (mainly psychometric) literature, and we discuss how actual psychotherapy researchers could think about and discuss the epistemic validity of (1) their operationalization and (2) its function in their overall epistemic process.

## Validity Beyond Psychometrics

In this section, we discuss the current use of validity terminology in psychological research, to understand how it fails to cover the problem of applied research that we elucidated in the previous section. First, we argue that the dominant understanding of *psychometric validity* is insufficient in covering the question of what constitutes a valid epistemic process in psychotherapy research. Subsequently, we overview the available terminology regarding *validity of research* to evaluate whether those terms allow for consideration of the validity of operationalization sequences in applied research, as well as the function of the chosen operationalizations within the epistemic endeavor of working researchers.

Validity became a field-wide issue in psychology when the American Psychological Association initiated a task force to work out guidelines for quality control of testing in psychology and education (Newton and Shaw, 2013, 2014; Slaney, 2017). This led to international *Standards for Educational and Psychological Testing*, which were initially based on the canonical paper by Cronbach and Meehl (1955). According to Cronbach, it is not only important to safeguard a measure’s ability to measure what is meant to be measured, but it is also crucial for test developers to provide guidelines for valid *test use*, so that test score interpretation can be accurately embedded in and justified by the current nomological network. Three decades later, Messick (1980, 1989) distinguished validity from the social consequences of use. According to Messick, an instrument should bear *construct* validity, which is the capacity of the test itself to capture the purported constructs or theorized variables, but this validity should be independent of the application of the measure in specific contexts. According to Messick, proper application is as important, but strictly speaking, it should not be called validity of tests, as the specific local consequences of application are not inherent to the test itself. Based on Messick’s argument, the term validity thus became separated from test use, and while Messick stressed the importance of both, his divide resulted in an increasing emphasis on validity as a test capacity rather than sound use or interpretation. This psychometric prioritizing of validity of tests is substantiated by guidelines drafted by influential institutions such as the US Food and Drug Administration (US Department of Health and Human Services Food and Drug Administration, 2009). Hitherto, the discussion still goes back and forth between “liberalists” (e.g., Crooks et al., 1996; Kane, 2013) who focus on a justified use and interpretation of test scores given a theoretical network in which interpretations are embedded (Newton and Shaw, 2014, p. 176 and onward), and “conservatists” (e.g., Lissitz and Samuelsen, 2007; Cizek, 2012; Borsboom, 2015) who argue that validity should be solely focused on the psychometric soundness of the test itself to capture its intended construct.

According to Borsboom et al. (2004), “the concept that validity theorists are concerned with seems strangely divorced from the concept that working researchers have in mind when posing the question of validity” (p. 1061). With ‘working researchers’, they refer to test developers that design the measures. For a test developer, they argue, every reference to theory, nomological networks, or embeddedness of interpretation in the then current body of scientific knowledge, would distract from his primary task to guarantee that the measure actually measures the real^13^ construct that it purports to measure (Borsboom et al., 2004, p. 1061). In the same line, Strauss and Smith (2009) emphasize the necessity of measuring unidimensional constructs for the sake of valid measurement, in which they understand “psychology” as experimental or lab psychology, which, however, is only one branch of the broad field of psychological research. By interpreting “the working researcher” as psychometric researchers or test developers, and “psychology” as limited to the experimental approach, the term validity becomes a psychometric concept that indeed should be as clear as possible for the researcher who works in test construction or experimental test research.

However, the problem is that this strictly psychometric interpretation prohibits a whole range of researchers in the fields of psychology and education from actually considering the validity of their use of tests in epistemic research. Importantly, the epistemic goal for applied researchers is principally different from the psychometric goal of the test developer (cf. Elliott and McKaughan, 2013; cf. footnote 3). This different, non-psychometric researcher was already addressed by Cronbach (1988), Kane (1992), and even Messick in his early years (see Newton and Shaw, 2014). They argued that flawed use of a test does decrease the validity of the test instrument itself, and thus the intended test use should be part of the *validation* of the test, which entails both the interpretation of score meaning and the ethical consequences of test interpretation. ^14^ Importantly, these arguments are still focused on guaranteeing the validity of the *instrument*, in which “test use” is understood as the application of the test *as* intended by the test developer.

Recently, a powerful argument was made by Moss et al. (2004) to broaden the term validity to *validity of action*, to enable users to validly apply and combine tests in assessment practice in ways that go beyond what test developers *can* intend to be tested. For example, teachers who administer several tests during the academic year to evaluate whether a student is ready to graduate, cannot simply rely on the validity of one test but need to validly combine sources of information to form a justified judgment (cf. Jukola, 2017, on judgment in standardized scientific assessment). In this educational setting, the validity argument needs to go beyond the psychometric properties of the test (cf. conservative view on psychometric validity), *and* beyond the nomological network in which the proposed construct of the test is embedded (cf. liberal view on psychometric validity), as it has to capture *the combination* of tests as input for valid judgment in a dynamical and (in this example) individualized situation.

The test use situation that Moss et al. (2004) refer to is similar to diagnostic practice of psychologists who, for example, combine multiple sources of information to assess patients’ psychopathological symptoms before admitting them to a treatment facility. Whereas Moss et al. (2004; see also Moss, 2013) make a cogent argument for the necessity of “validity in action”, our paper is not focused on test use in clinical or educational practice but in clinical research, in which the “working researcher” is the researcher who conducts clinical or psychotherapy research within a specific epistemic framework and with a specific epistemic goal. In the context of psychotherapy research, the epistemic goal is not to indicate the presence and severity of symptoms *per se*, but to interpret the scores as a signal of something else. In this context, the instrument is used for a different target than it was designed for; that is, it is applied in a different research context with a different – often broader – goal than just measuring a certain construct. Therefore, we do not only go beyond validity of tests but also beyond validity of testing as an action of assessment; we address the overarching validity of the research process in which testing can be used *as part of* the broader epistemic endeavor of the researcher.

### Validity of Research

To be able to discuss the validity of the research process, Campbell (1957) proposed the term “internal validity”, referring to the soundness of the experimental design. In the context of test construction, internal validity refers to the association between items within scales as related to the overall measure. But according to Campbell, internal validity can also be used to evaluate whether the factors (both the constructs and the operationalizations) and their relations that are proposed in an experimental research design, indeed allow for a sound conclusion. For example, if a researcher intends to draw causal conclusions based on his research, it is necessary to use some sort of interventionist design (cf. Woodward, 2003) that indeed allows for causal conclusions, such as randomized controlled designs (RCTs, cf. Kazdin, 2008 and Desmet, 2013, for a discussion of this design in psychotherapy research).

When interpreted as a concept of “validity of research”, “internal validity” could indeed cover the validity of the sequence of operationalizations that was discussed in the previous section. However, as we pointed out before, even when researchers consider the validity of their research within their epistemic proceedings, there is still little opportunity to critically discuss regarding validity issues within the strictly outlined IMRAD publication format. As the IMRAD model heuristically places validity under the Measures header in the Methods section, it implies an instrument-focused consideration of validity that does not allow for proper consideration of epistemic choices or practical and epistemic problems that researchers encounter in designing and conducting the research design.^15^ Importantly, as the IMRAD model does not allow for such a discussion, the considerations are relegated to conceptual or scientific opinion papers. This is not sufficient because it limits dialog amongst working researchers on the concrete epistemic issues they face in *doing* the research – and it also gives the impression that published empirical papers are free from validity issues in the overall procedure (a conclusion that would thus be derived by means of face validity). Therefore, to be able to accurately discuss validity of research in psychological papers, the IMRAD model should be broadened (or *loosened*) to stimulate the consideration of internal validity of research issues that are relevant to “working researchers” in psychotherapy research.

Although a proper dialog on issues of internal validity would vitally aid valid psychotherapy research, it is important to notice that the idea of internal validity is building on a notion of realism, as it implies that given a certain specified goal, there can be one right way of doing research (cf. Slaney, 2017, for a discussion of the status of realism in validity debates). However, the fact that a design can have internal validity does not imply *whether* the researcher should indeed choose this design to answer his epistemic research questions. A chosen design may be valid as a means to satisfy the intended goal, but that does not imply that it is the only nor the most appropriate means that the researcher could choose.^16^ In practice, researchers can choose multiple research designs, using multiple operationalizations and assessment methods. Consequently, the design is not an epistemic given, but a pragmatic, contextual and human-made choice that is informed by the researcher’s epistemic framework and scientific goals (cf. Elliott and McKaughan, 2013). Importantly, the researcher’s *choice for* a design as a means to answer his or her specific epistemic question is not accounted for by internal validity (Figure 5).

Moreover, it is not covered by the “external validity” that was proposed by Campbell (1957) either. External validity refers to the generalizability or applicability of results and/or conclusions to population level. Consequently, it only covers validity of the research *product*, but not the choice for the research based on the researcher’s epistemic aim *per se*. This is better covered by a branch of external validity that is known as Ecological Validity, which means that the research set-up resembles daily life situations (cf. Brewer and Crano, 2000). Although this surely is an important consideration regarding validity of research, it is just one type of consideration in the range of decisions to be made in the entire research endeavor. Mook (1983) even argues that it is up to the researcher to decide to what extent he or she thinks it is appropriate to generalize findings to populations or daily life situations, depending on the specific research goals. According to Schmuckler (2001), “one problem with this multidimensionality, however, is that no explicit criteria have been offered for applying this concept [of ecological validity] to an evaluation of research” (p. 419; see Schmuckler, 2001, for a historical overview of the various modalities of the term ecological validity).

The concepts internal, external and ecological validity thus do not (clearly) cover the entire scope of the research procedure, not even when combined. Moreover, this multitude of types of validity that working researchers can take into account, may give the impression that researchers can pick and choose whichever type they value most within the context of their research endeavor (cf. Mook, 1983). Yet the fact *that* researchers can pursue such choices, show that researchers have to make choices on the value and direction of their research even before and beyond choosing sound and valid methods. The entirety of epistemic choices within research set-up, is and should be subject to validity questioning.

This brings us to the validity of the *function* of operationalization in applied research. As the function of the design is bound to the epistemic proceedings of the researcher within a specific scientific and societal context, its validity could not be stated *a priori* nor context-independent, which makes the realist notion of internal validity insufficient to capture the validity of the overall epistemic endeavor (cf. Hacking, 1983). To illustrate the importance as well as the non-self-evidence of this function, consider the following example. EBT is often justified as a way to offer the most effective treatment to the largest amount of people. For such a goal, it does not *necessarily* make epistemic sense to use a symptom measure such as the BDI, as finding that people cry less than before after a course of therapy does not imply more working days or less sick leave, for example. So, if the epistemic goal were to scrutinize the proportion of patients that would actually function better after treatment in a societal sense, it could be more utile to measure “efficacy” by means of sick leave days than by use of the BDI. If the goal were to scrutinize the amount of people that do not relapse, which requires durability of changes that were brought about during treatment, it would make more epistemic sense to measure specific dysfunctional experiences. And if the goal were to reduce the risk on long-term health care costs, it would be reasonable to indicate treatment success by means of long-term cortisol level monitoring.

Importantly, thus, the specific end that a researcher intends to satisfy by his epistemic endeavor should be specified in order to evaluate whether an outcome measure can function validly as a means to indicate the target concept. This indeed goes beyond the realist notion of internal validity of the design that was discussed before, as the target concept could be validly operationalized in different ways, but the choice for one of those many operationalizations should be arguably appropriate to satisfy the actual epistemic goal of research. As the validity of this function of research goes beyond the validity of the operationalization sequence in the design itself, it is vital for valid psychotherapy research to be able to consider the overall validity of this function of the chosen research procedure (cf. Westen et al., 2004).

To enable researchers to consider the validity of this specific, local, and practical function of the design within their epistemic endeavor, we use the working concept *validity of the epistemic process*, or – in short – epistemic validity. We use this term purely for the sake of our argument, to signal the issue of validity for the *overall epistemic process* that is involved before and beyond the practical operationalization that is heuristically considered to be at stake when validity is considered. This term is used to demarcate it clearly from psychometric validity that covers parts of the *operationalization* within research. Further, as internal, external and ecological validity all “start” from the chosen design, but do not capture *whether* a design is valid given its function within the overall epistemic process, we chose epistemic validity over the previous terms associated with validity of research.

This broad notion of validity of the overall epistemic process is close to the principle of methodological quality that Levitt et al. (2017; APA *Task Force on Resources for the Publication of Qualitative Research*) have formulated for qualitative research. In an effort to summarize diverse terms used in the field of quality control, they propose the use of the term Methodological Integrity as the operationalization of trustworthiness of research, which they define as follows:

“Integrity is the aim of making decisions that best support the application of methods, as evaluated in relation to the following qualities of each study. Integrity is established when *research designs* and *procedures* […] support the *research goals* (i.e., the research problems/questions); respect the researcher’s *approaches to inquiry* (i.e., research traditions sometimes described as world views, paradigms, or philosophical/epistemological assumptions); and are tailored for *fundamental characteristics of the subject matter and the investigators*”. (Levitt et al., 2017, pp. 9–10; italics in original).

Levitt et al. (2017) define integrity as composed of two flexible criteria that allow for assessment of the trustworthiness of the very diverse types of qualitative research and within varied or even contrasting epistemic modes. First, *fidelity* concerns “the intimate connection that researchers can obtain with the phenomenon under study; […] regardless of whether [researchers] view the phenomena under study as social constructions, existential givens, unmediated experiences, embodied practices, or any kind of subject matter that may be reflected in data and analyses” (Levitt et al., 2017, p. 10). Second, *utility* concerns the “effectiveness of the research design and methods, and their synergistic relationships, in achieving study goals; […] i.e., method as useful toward what end?” (ibid.) – which the authors emphasize to argue against a de-contextualized consideration of methods and procedures.

The formulations and aims of this task force indeed are close to the aim that we set out in this paper. To make sure that validity of research is considered as at least as important as validity of measurement, however, we deem it important to acknowledge that these issues together still regard the *validity* of research. Terms such as integrity, coherence, trustworthiness, fidelity, and utility, that are promoted by these and other qualitative researchers in psychology (cf. Stiles, 1993; Elliott et al., 1999; Kvale and Brinkmann, 2009), cover a lot of our concerns, but they do not signal the validity root as firmly, whereas all proposed quality control concepts in qualitative research in fact fall under the umbrella of validity of the overall research process (cf. Newton and Shaw, 2013).

Moreover, whereas the term integrity suggests a solo enterprise bound to specific studies (cf. internal validity), epistemic validity also captures more general discursive problems in the psychological field (see also Prilleltensky, 2008), such as the issues that were listed in the introduction, which share a common root in the overall validity of research. Importantly, also the initial consideration of applying quality control based on a qualitative or quantitative research paradigm *per se* falls under the validity of the entire research endeavor. This way, our use of the term validity goes beyond semantics: epistemic validity may be considered the umbrella term that captures the qualitative concepts of research integrity as well (see also footnote 12). It is not necessary to use this exact term, yet it is crucial that the used term enables researchers to denote their own epistemic stance within their scientific endeavor. We call this necessary because with the current emphasis on EBT, research increasingly influences practice, and in every step down the line from research to dissemination to practice, the idea of validity becomes more heuristic, which gives the impression that research is “right”. That said, it seems crucial that researchers themselves ask the question of validity of their means within their epistemic approach, as they may be ascertained that people in practice – e.g., patients, health care workers, and policy makers – will ascribe a certain truth value to them (cf. Douglas, 2009).

## Conclusion

In this paper, we argued that the default psychometric understanding of “validity” in psychology is insufficient in capturing all the validity issues involved in the epistemic process of psychotherapy research. In the first section, we used the example of the BDI to show that reliance on psychometric validity does not guarantee a valid psychotherapy research at large. Surely, we are not the first to make this argument, but given the persistently limited consideration of validity under the Measures header in empirical psychological research papers, we deem it necessary to show this problem in the most concrete terms, so that our argumentation is as close as possible to the concrete decisions that are made daily by working psychological researchers. As we noted in this paper, we do not believe that epistemic validity is never considered by psychotherapy researchers, but given the prominent psychometric interpretation that is substantiated by the format limitations in the IMRAD model, validity is too often just discussed *as if* it were test validity (e.g., Newton and Shaw, 2014, p. 9 and onward; see footnote 15).

As we argued that test validity is too limited to account for the overall epistemic validity of the research procedure in psychotherapy research, we conclude that it would not be epistemically valid to rely on test validity for the entire procedure, not even heuristically. Especially in times in which the emphasis on EBT is increasing exponentially and quantitative research methods are discursively prioritized, psychotherapy researchers should at least ask the question of validity of their preferred research methods as means to satisfy their epistemic and/or clinical goals. Therefore, it is necessary to think carefully about what the goal is concretely, to be able to analyze the validity of the chosen means within the overall epistemic procedure. That is, it seems crucial that researchers themselves ask the question of validity of their means within their epistemic approach, to be able to validly derive “evidence” for EBTs in psychotherapy.

## Data Availability

All data are available upon request. Data are anonymized according to the privacy considerations that were formalized in the informed consent form that was signed by each participant in the study.

## Author Contributions

This paper was a joint effort by FT, SC, MD, MDS, and RM. Data was collected as part of a broader psychotherapy study, conducted by a research team at the Department of Psychoanalysis and Clinical Consulting, Ghent University, Belgium. MD was involved as therapist in the phase of data collection, and MD and RM as supervised the project (“SCS”). SC, MDS, and FT were involved in data collection and management and conducted interviews with the patient-participants. SC and MD carried out an evidence-based intrinsic case study on the data of the patient. FT discussed the findings with SC, MDS, and MD and reinterpreted the available data in the context of methodological conduct. FT developed the validity argumentation, in which the case serves as an exhibit. MDS audited the validity interpretations and contributed to the manuscript revision.

## Conflict of Interest Statement

The authors declare that the research was conducted in the absence of any commercial or financial relationships that could be construed as a potential conflict of interest.

## Footnotes

1 Note that the expression “valid measures” is technically misleading as no instrument is valid in itself: instruments can only be used validly in a specified domain of application and with guidelines on score interpretation (Newton and Shaw, 2014).

2 In this paper, we distinguish validity of instruments from the validity of the overall scientific endeavor. We refer to the validity of instruments either by the term “test validity”, “instrumental validity”, or “psychometric validity”. We refer to the validity of the overall scientific endeavor by the term “epistemic validity”.

3 Note that the level of goal-orientedness may differ *within* scientific disciplines as well. Psychology is exemplary, as psychotherapeutic and clinical research are explicitly focused at providing evidence for *use* in clinical practice, whereas branches of experimental psychology, for example, are focused on gathering evidence for the sake of knowledge expansion *per se*. This difference in goals affects the requirements for research methodology (cf. Mook, 1983), as the more fundamental subdisciplines hold stricter requirements for standardization and control and value generalizability less, while for the more applied subdisciplines it is vice versa.

4 For a thorough discussion of the feasibility of “construct validity”, see Newton and Shaw (2014), Alexandrova and Haybron (2016), and Slaney (2017).

5 Note that in the sequence of operationalizations that is used in a RCT-type pre-post design, it is necessary to operationalize efficacy in terms of change, which means that the BDI is not only used to signal another construct (i.e., treatment success) but also that a BDI difference score is necessary to indicate change over time. The use of a difference score as indicative for change over treatment is an operationalization in itself, thus it should be possible to judge this step on its validity. See Westen et al. (2004) and McClimans (2010), for example, on the assumption of stable numerical representation of therapeutic transformation.

6 Note that “treatment efficacy” can only formally be substantiated if treatment success is shown in at least two independent randomized controlled trials (Chambless and Hollon, 1998). Therefore, in this section we continue our argument with the concept “treatment success”.

7 All data used in this paper was previously published by Cornelis et al. (2017). Tables are reprinted with permission.

8 The study design and proceedings were approved by the Ethical Board of the Ghent University Hospital in Belgium (Registration no. B670201318127). All patients gave written informed consent to collect, analyze, and publish their individual data throughout and after treatment. All identifying information concerning the patient has been changed to protect confidentiality. The data are denoted by an anonymous participant code and the patient is referred to by a pseudonym.

9 In the BDI guidelines, the following classification is given to determine the severity of depression based on BDI-scores: 0–13: minimal depression, 14–19: mild depression, 20–28: moderate depression, or 29–63: severe depression (Beck et al., 1996).

10 In the on-going discussion on construct validity regarding test construction and use, different authors disagree on whether it should be assumed that the test does capture an ontological essence of the purported construct. The tension between realists (e.g., Borsboom et al., 2004) and instrumentalists (e.g., Cronbach and Meehl, 1955; Kane, 2013) is described extensively in Newton and Shaw (2014; Chapter 5). However, the argument in the current paper is not focused on the target construct of the measure but on the construct that is represented by another construct, which in this example could convey multiple (moral, societal, clinical, technical…) interpretations. Therefore, the discussion on ontology of construct measures does not apply to our examples, and subsequently the discussion does not allow for a primary solution to the problem that we propose in this paper. See further the next section of this paper.

11 Another example of where validity problems go beyond the scope of instrumental validity *per se*, regards the content of data which is “collected from” sense-making agents, i.e., human beings that wonder why they are assessed or participate in assessment with a concrete motivation to be assessed. Elaborating on this issue would be beyond the scope of the current paper, so we refer to Truijens et al. (2019), which provides a detailed analysis of validity problems that appear at the level of “the data” in psychotherapy research, but *cannot* be taken into account by means of psychometric validity.

12 We use the term epistemic validity to denote the *validity of the overall epistemic process* as a working concept, to make clear that different validity issues share a common root in the overall epistemic process. The term “epistemic validity” has been used before in different contexts. Prilleltensky (2008) proposed it as one of two parts of “psychopolitical validity”. According to Prilleltensky (2008), epistemic psychopolitical validity “is achieved by the systematic account of the role of power in political and psychological dynamics affecting phenomena of interest” (p. 130), whereas the “transformative” part is focused on the social changes that are brought about by these power relations. Based on these terms, he argues for the need of incorporating and evaluating power relations in knowledge generation practices, within the broader initiative of action psychology or power psychology. We consider these issues important indeed, but rather than using “epistemic validity” to focus specifically on power relations in the community, we take power relations as one possible part of the overall epistemic process, as this process indeed includes multiple levels of decision making that are issue to validity questions. Further, the term epistemic validation is used by Costa and Levi (1996) to evaluate whether “causal conditionals” (i.e., factors that are hypothesized to be causal, within a theoretical framework) can be made concrete within testable “epistemic models” (rather than just be theoretically argued for) and therefore can be evaluated on their truth value in concrete terms. Here, the term epistemic validation serves the purpose of evaluating the logic of formal arguments in concrete terms, yet the content of the epistemic models is not (and does not have to be) made concrete for these authors. For our purposes, though, we use to term precisely to signal those concrete epistemic circumstances that are encountered in applied research.

13 See Slaney (2017) for a thorough discussion of the status and developments of realism in scientific thinking, and how that relates to the understanding of validity.

14 Borsboom (2006) would in response argue that validation is not relevant to the question of validity of measures as there cannot be such a thing as a “level of validity”: according to Borsboom, a measure either is valid, or it is not. This is based on a strictly realist ontological premise, which, however, seems hard to defend in applied social science (cf. Gergen, 2001; Alexandrova, 2016; Alexandrova and Haybron, 2016).

15 Such ‘heuristic’ understanding (cf. Hathcoat, 2013) is also noticeable in the recent book “validity in psychological and educational assessment” by Newton and Shaw (2014) that explicitly addresses working researchers. In their introduction, they distinguish between “validity of measurement” and “validity of research” (p. 9 and onward), and remark that when they use “validity”, they mean “validity of measurement”. This heuristic use of validity terminology echoes our earlier point that by interpreting “the working researcher” as a psychometric researcher (Borsboom, 2006) and “psychology” as experimental psychology (Strauss and Smith, 2009), validity will be kept hostage in a psychometric debate that is not sufficient to cover the validity of applied research as a scientific endeavor.

16 See Cartwright and Stegenga (2011) for a discussion on sufficient but unnecessary conditions (“INUS-conditions”) to derive evidence that is useful and valid in practice. Their discussion is focused at decision making in function of evidence-based policy, but their use of INUS-conditions provides an insightfull framework to understand the decision on the appropriateness of methods and definition of evidence in applied research as well.
